# Analysis and Functional Verification of *PlPM19L* Gene Associated with Drought-Resistance in *Paeonia lactiflora* Pall.

**DOI:** 10.3390/ijms232415695

**Published:** 2022-12-10

**Authors:** Jiasong Meng, Jinhui Guo, Tingting Li, Zijie Chen, Miao Li, Daqiu Zhao, Jun Tao

**Affiliations:** 1 College of Horticulture and Landscape Architecture, Yangzhou University, Yangzhou 225009, China; 2 Joint International Research Laboratory of Agriculture and Agri-Product Safety, The Ministry of Education of China, Yangzhou University, Yangzhou 225009, China

**Keywords:** *Paeonia lactiflora*, membrane protein, *PM19L*, drought-resistance

## Abstract

The herbaceous peony (*Paeonia lactiflora* Pall.) is widely cultivated as an ornamental, medicinal and edible plant in China. Drought stress can seriously affect the growth of herbaceous peony and reduce its quality. In our previous research, a significantly differentially expressed gene, *PM19L*, was obtained in herbaceous peony under drought stress based on transcriptome analysis, but little is known about its function. In this study, the first *PM19L* that was isolated in herbaceous peony was comprised of 910 bp, and was designated as *PlPM19L* (OP480984). It had a complete open reading frame of 537 bp and encoded a 178-amino acid protein with a molecular weight of 18.95 kDa, which was located in the membrane. When *PlPM19L* was transferred into tobacco, the transgenic plants had enhanced tolerance to drought stress, potentially due to the increase in the abscisic acid (ABA) content and the reduction in the level of hydrogen peroxide (H_2_O_2_). In addition, the enhanced ability to scavenge H_2_O_2_ under drought stress led to improvements in the enzyme activity and the potential photosynthetic capacity. These results combined suggest that *PlPM19L* is a key factor to conferring drought stress tolerance in herbaceous peony and provide a scientific theoretical basis for the following improvement in the drought resistance of herbaceous peony and other plants through genetic engineering technology.

## 1. Introduction

The herbaceous peony (*Paeonia lactiflora* Pall.), a perennial herbaceous flower of Paeoniaceae, is regarded as ‘the Prime-Minister of flowers’ in China. It has been cultivated as a famous flower for more than 4000 years, and it is widely distributed in China [1]. It is widely loved in China because of its large flower, diverse color and graceful gesture, and it is widely cut and potted, and used as an ornamental plant in specific gardens and urban green spaces in China [2]. The herbaceous peony was originally cultivated as a traditional Chinese medicine ‘Radix Paeoniae Alba’ in China [3]. However, in recent years, as a novel cut flower, it has become important in the international market [4]. The seeds of the herbaceous peony are composed of approximately 25% oil, with more than 90% of this being made up by unsaturated fatty acids, which can also be used as an edible oil resource [3]. The herbaceous peony prefers a cool climate, and growers should avoid exposing these plants to high temperatures and humidity levels. They can be grown successfully in temperate, cold-winter climatic zones (generally USDA cold hardiness zones 3–8) [5], which include to the northern region of China, including Shandong, Henan, Hebei, Shanxi, Qinghai, Liaoning, Jilin, etc. [6]. While the northern region of China is mostly arid or semi-arid, the climate is generally dry because there is slight precipitation in spring, and the summer is hot and rainless. In addition, during the growing period, the soil herbaceous peonies are planted in should be kept moist, and in particular, it must not be arid before the flowering period, or the plants can easily wihter [7]. Obviously, the herbaceous peony has a certain ability to withstand drought, but long-term drought and severe shortages of water will threaten its normal growth and development [8]. Moreover, in recent years, the area of land that is classed as semi-arid has increased rapidly in China [9], as the temperature has increased significantly with small fluctuations, and the average precipitation level has decreased considerably [10]. Therefore, drought is the main abiotic stress factor that affects the cultivation of herbaceous peony in northern China.

Drought is a severe and complex type of abiotic stress which has adverse effects on plant growth, development, yield, ornamental quality, etc. Understanding the regulation mechanism of plants’ responses to drought stress will help improve the plants’ tolerance to drought stress [11,12]. When plants experience drought stress, the signal system in response to drought stress will be activated, and the expressions of drought-related genes are regulated such as the ABA-responsive element gene (*ABRE*) [13], dehydration-responsive element binding gene (*DREB*) [14], Really Interesting New Gene zinc finger protein 1 (*RINGzf1*) [15], etc. These genes participate in the whole process of the stress response, including signal transduction, transcriptional regulation, the preservation of membranes and proteins, the scavenging of reactive oxygen species, etc. [16]. Plants can recognize shortages of water at their roots and transmit a signal to their shoots to synthesize abscisic acid (ABA) in their leaves. ABA, a kind of phytohormone, is a key regulator of plants’ physiological and molecular responses to drought stress, such as stomatal closure, gene expression, and the accumulation of osmoprotectants and stress proteins [17]. When exposed to drought stress, ABA in plants accumulates extensively, leading to the stomatal closure of leaves, which is one of the most typical characteristics of the physiological response to cope with drought in plants [18]. Postinglion and Muday [19] suggested that ABA promotes the synthesis of H_2_O_2_ in guard cells by binding NADPH oxidase with the membrane; then, H_2_O_2_ mediates stomatal closure by activating (hyperpolarized) plasma membrane Ca^2+^ channels. Therefore, ABA plays an important role in the process of the response to drought stress.

Previous reports demonstrated that the expression of *Plasma Membrane 19-Like* (*PM19L*) genes was associated with dormancy and the ABA hormone [20,21]. The PM19L protein belongs to the AWPM19 protein family, which was first reported in wheat and is exactly identical to ABA-induced the 19-kDa plasma membrane polypeptide [22]. The AWPM19 family proteins are highly correlated with embryo development, seed germination and dormancy, and responses to environmental stress such as drought [23]. Previous studies have shown that in rice, the *OsPM19L1* gene belonging to the AWPM19 protein family participated in the response to drought, high-salt and low-temperature stress through the ABA signaling pathways induced by stress [20]. At present, the *PM19L* gene has been isolated from rice [20], *Arabidopsis* [21], wheat [22], barley [23], etc. In addition, our previous studies showed that the expression level of the plasma membrane protein *PM19L* gene was significantly different based on the transcriptome analysis of *P. lactiflora* under drought stress [8]. However, the characteristic and function of the *PM19L* gene in *P. lactiflora* is unclear. In the present study, we cloned the *PM19L* gene, analyzed its sequence, observed the subcellular localization and investigated the function by overexpression in *Nicotiana tabacum* L. under drought stress. Our research lays the foundation for the further identification and application of the *PlPM19L* gene in the molecular genetic improvement of herbaceous peonies’ tolerance to drought.

## 2. Result

### 2.1. Gene Clone and Sequence Analysis of PlPM19L from P. lactiflora

**Figure 1:**
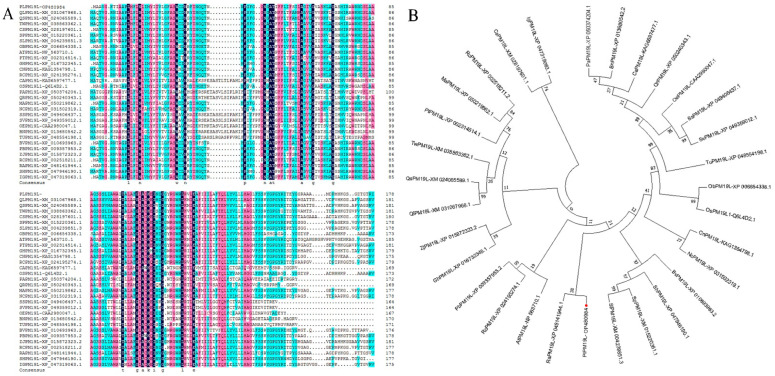
Sequence analysis of *PlPM19L.* (**A**) Alignment of protein sequence of PlPM19L (OP480984) and other plant species. (**B**) Phylogenic analysis of *PM19L* in *Paeonia lactiflora* and other plant species. Other plant species including *Quercus lobata* (Ql), *Quercus suber*, *Tripterygium wilfordii* (Tw)*, Camellia sinensis* (Cs), *Solanum pennellii* (Sp), *Solanum lycopersicum* (Sl), *Oryza brachyantha* (Ob), *Arabidopsis thaliana* (At), *Populus trichocarpa* (Pt), *Gossypium hirsutum* (Gh), *Cocos nucifera* (Cn), *Rosa chinensis* (Rc-XP_024195274.1), *Cucurbita argyrosperma* subsp. *sororia* (Cr), *Oryza sativa* (Os), *Potentilla anserina* (Pa), *Quercus robur* (Qr), *Mercurialis annua* (Ma), *Nymphaea colorata* (Nc), *Solanum stenotomum* (Ss), *Solanum verrucosum* (Sv), *Olea europaea* subsp. *europaea* (Oe), *Brassica napus* (Bn), *Triticum urartu* (Tu), *Beta vulgaris* subsp. *vulgaris* (Bv), *Pyrus* × *bretschneideri* (Pb), *Ziziphus jujuba* var. *spinosa* (Zj), *Ricinus communis* (Rc-XP_002518211.2), *Rhodamnia argentea* (Ra), *Salvia hispanica* (Sh) and *Impatiens glandulifera* (Ig).

Specific primers were designed according to the fragment sequence of the *PM19L* gene (ID: Unigene0041970) from the transcriptome database (SRP131648) [8] and applied to clone the gene named *PlPM19L* via the rapid amplification of cDNA ends (RACE) from *P. lactiflora* ‘Da Fugui’, which is main variety of cut peony in China. The total length of the *PlPM19L* gene is 910 bp, and it has 537 bp open reading frames and encodes 178 amino acids (the accession number is OP480984). After analysis with the ProtParam online tool, the isoelectric point of *PlPM19L* was found to be 9.79, and the molecular weight of *PlPM19L* was found to be 18.95 KDa. It is worth noting that the instability index (II) of the PlPM19L protein was computed to be 22.73, and the PlPM19L protein was classified as a stable protein. Moreover, the grand average of hydropathicity (GRAVY) of the PlPM19L protein was 0.594, and it was a hydrophobic protein. In addition, the sequence alignment compared the amino acid sequence of PlPM19L with the amino acid sequence of other plants in the NCBI via DNAMAN. It was found that they had certain similarities (Figure 1A). Subsequently, a phylogenetic tree was constructed with the neighbor-joining method using MEGA 7.0 following multiple alignments of protein sequences with ClustalW (Figure 1B). The alignment results indicated that the PlPM19L protein sequence has consistency in different plants and the PlPM19L protein has the highest evolutionary similarity with *Rhodamnia argentea* (XP_048141944.1).

### 2.2. Expression Level Analysis of PlPM19L in P. lactiflora

**Figure 2:**
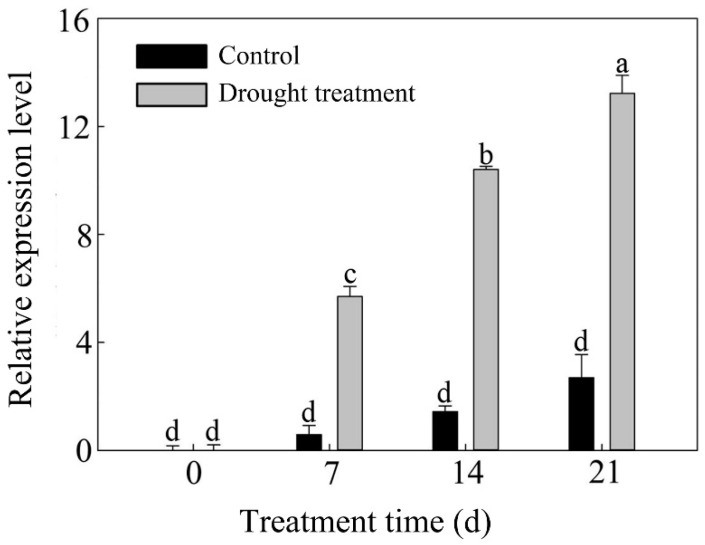
Expression level analysis of *PlPM19L.* Values are the means ± SE of three replicates carried out on cDNAs obtained from three independent mRNA extraction. Different letters indicate significant differences (*p* < 0.05), and the same letters indicate no significant differences.

In order to detect the expression of *PlPM19L* in the leaves of *P. lactiflora* ‘Dafugui’ at different stages after treatments and the blank control, quantitative real-time polymerase chain reaction (qRT-PCR) was used. *PlActin* (JN105299) was used as an internal reference gene [24]. As shown in Figure 2, in both the control and drought stress treatments of *P. lactiflora* ‘Dafugui’, the expression level of the *PlPM19L* gene in the leaves increased with the development of the leaves. Compared with the control, the expression level of the *PlPM19L* gene was significantly up-regulated under drought stress.

### 2.3. Subcellular Localization of the PlPM19L Protein

**Figure 3:**
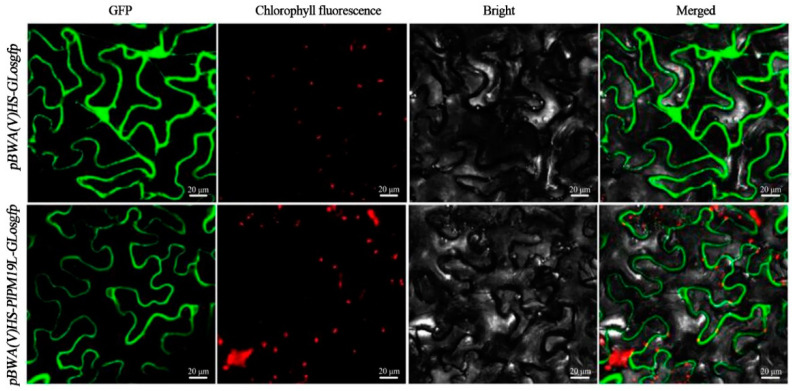
Subcellular localization of the PlPM19L protein in tobacco. An empty vector *pBWA(V)HS-GLosgfp* and *pBWA(V)HS-PlPM19L-GLosgfp* transiently expressed in *N. tabacum* leaves. Bars = 20 μm.

To further determine the subcellular localization of the PlPM19L protein, the expression vector *pBWA(V)HS*-*PlPM19L-GLosgfp* was constructed and transformed into *Nicotiana tabacum* L. via the Fast Agro-mediated Seedling Transformation (FAST) method [25] using an empty vector, *pBWA(V)HS-GLosgfp*, as a control. As shown in Figure 3, the GFP green fluorescence sites were widely distributed in the whole plasma membrane system and nucleus in the pMDC43 empty vector. However, the GFP green fluorescence sites of *pBWA(V)HS*-*PlPM19L-GLosgfp* were located at the plasma membrane, indicating that PlPM19L was a membrane localization protein and consistent with the prediction result of subcellular localization.

### 2.4. Identification of Transgenic PlPM19L in N. tabacum

**Figure 4:**
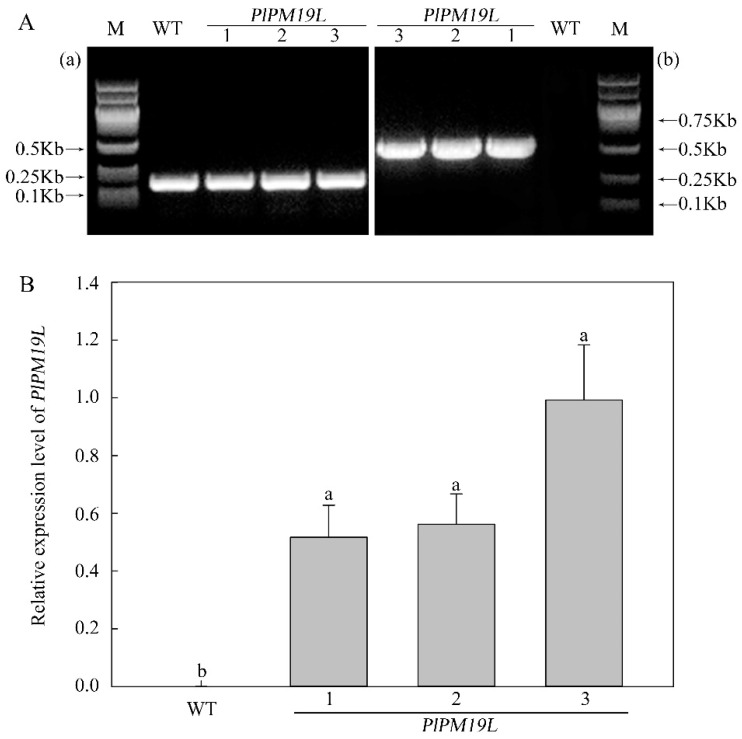
Identification of *PlPM19L* transgenic tobacco using PCR (**A**) and expression level analysis of *PlPM19L* in *N. tabacum* (**B**). (**a**) *NtActin* amplification tape; (**b**) *PlPM19L* gene amplification tape; M: DL 2000 Maker, WT: wild-type tobacco; 1, 2 and 3: transgenic tobacco. Values are the means ± SE of three replicates carried out on cDNAs obtained from three independent mRNA extraction. Different letters indicate significant differences (*p* < 0.05), and the same letters indicate no significant differences.

Wild-type tobacco and transgenic tobacco were cultured under the same conditions for 60 days and then subjected to natural drought stress treatment for 10 days. DNA was extracted from appropriate leaves, and PCR amplification was performed. The results showed that the *PlPM19L* gene was not detected in wild-type tobacco, but a single bright clear band was amplified in transgenic tobacco (Figure 4A). The qRT-PCR results showed that the expression level of *PlPM19L* in transgenic tobacco was significantly higher than that in wild-type tobacco (Figure 4B), indicating that the overexpression vector *pBWA(V)HS-PLPM19L-GloSGFP* had been successfully introduced into tobacco.

### 2.5. Effects of Drought Stress on the Phenotype of Transgenic PlPM19L in N. tabacum

**Figure 5:**
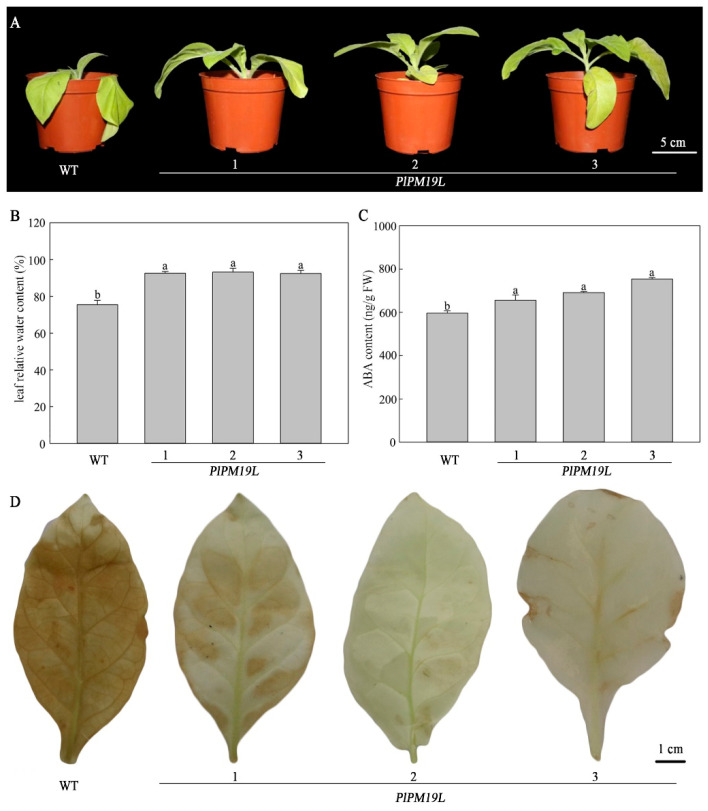
Phenotypic observation and physiological indices of *PlPM19L* transgenic tobacco under drought stress. (**A**) Phenotype. (**B**) Relative water content. (**C**) ABA content. (**D**) H_2_O_2_ accumulation. WT: wild-type tobacco; 1, 2 and 3: transgenic tobacco. Values are the means ± SE of three replicates. Different letters indicate significant differences (*p* < 0.05), and the same letters indicate no significant differences.

The leaf is the main organ for photosynthesis and transpiration, it is the main channel of water exchange, and it is sensitive to environmental changes. Under drought stress, in the process of water loss in plants, leaves suffer different degrees of dehydration, which lead to stomatal closure and limited photosynthesis, and even lead to damage to the photosynthetic structure and a serious decline in photosynthesis. Phenotypes of leaves from wild-type and transgenic tobacco treatment under natural drought stress were observed. As shown in Figure 5A, wild-type tobacco leaves wilted and drooped significantly, while transgenic tobacco leaves maintained a normal growth state on the 10th day of drought stress. The results showed that transgenic *PlPM19L* tobacco had stronger drought tolerance than wild-type tobacco from the plant phenotype.

### 2.6. Effects of Drought Stress on the Physiological Indices of Transgenic PlPM19L in N. tabacum

Water is one of the main factors affecting plant photosynthesis, and leaf water content will significantly decrease under drought stress conditions. Compared with wild-type tobacco, the leaf relative water content of the transgenic *PlPM19L* gene in tobacco was significantly higher under the drought stress treatment (Figure 5B). Under drought stress conditions, the endogenous ABA content in plants was increased to improve their own defense mechanisms. As shown in Figure 5C, the endogenous ABA content of transgenic tobacco was higher than that of wild-type tobacco under the same drought stress treatment. Hydrogen peroxide (H_2_O_2_) is involved in the regulation of a plant’s defense system to stress response and can enhance the tolerance of plants to abiotic stress. However, high concentrations of H_2_O_2_ will harm cells. Compared with wild-type tobacco, the transgenic tobacco leaves were lighter in color, indicating lower H_2_O_2_ accumulation in transgenic tobacco plants (Figure 5D).

### 2.7. Effects of Drought Stress on the Antioxidant Enzyme Activity of Transgenic PlPM19L in N. tabacum

**Figure 6:**
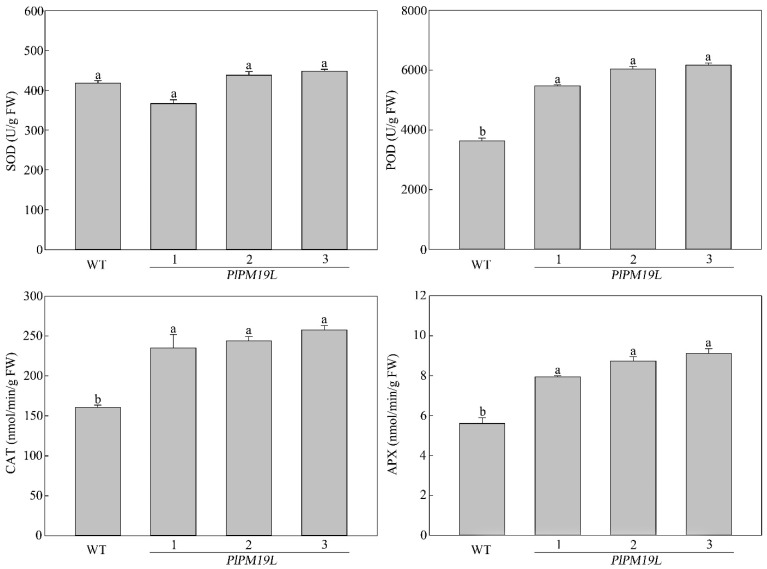
Protective enzyme activity of *PlPM19L* transgenic tobacco under drought stress. WT: wild-type tobacco; 1, 2 and 3: transgenic tobacco. Values are the means ± SE of three replicates. Different letters indicate significant differences (*p* < 0.05), and the same letters indicate no significant differences.

Superoxide dismutase (SOD), peroxidase (POD), catalase (CAT), and ascorbate peroxidase (APX) are the main antioxidant enzymes in plants, which can remove H_2_O_2_, superoxide anions, and hydroxyl radicals in plants; reduce the accumulation of reactive oxygen species; and resist the damage of plant cells. We measured the antioxidant enzyme activity of the drought-treated wild-type and transgenic tobacco (Figure 6). Compared with wild-type tobacco, the POD, CAT, and APX activity levels in transgenic tobacco leaves were significantly higher. The mean values of POD, CAT, and APX in transgenic *PlPM19L* tobacco were 1.62, 1.53 and 1.53 times those in wild-type tobacco, respectively. However, there was no significant difference in superoxide dismutase (SOD) activity between wild-type tobacco and transgenic tobacco. The results showed that the antioxidant enzyme activity levels in transgenic *PlPM19L* tobacco were higher than those in wild-type tobacco, indicating that transgenic *PlPM19L* tobacco is more tolerant to drought.

### 2.8. Effects of Drought Stress on the Photosynthetic Characteristics of Transgenic PlPM19L in N. tabacum

**Figure 7:**
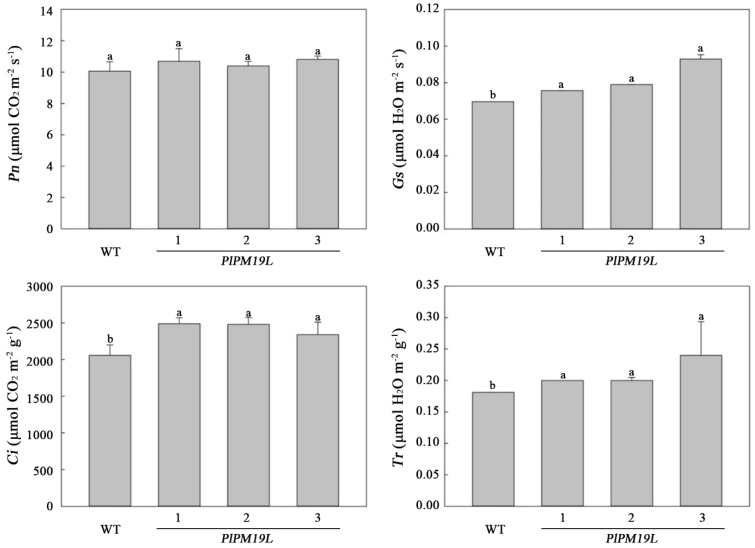
Photosynthetic characteristics of *PlPM19L* transgenic tobacco under drought stress. WT: wild-type tobacco; 1, 2 and 3: transgenic tobacco. Values are the means ± SE of three replicates. Different letters indicate significant differences (*p* < 0.05), and the same letters indicate no significant differences.

The process of photosynthesis is very sensitive to drought stress. In order to determine the difference in photosynthesis between transgenic *PlPM19L* tobacco and wild-type tobacco, a portable photosynthesis system (LI-6400, Li-Cor, Lincoln, NE, USA) was used to assess the photosynthetic characteristics. Compared with wild-type tobacco, the net photosynthetic rate (*Pn*), stomatal conductance (*Gs*), transpiration rate (*Tr*), and intercellular CO_2_ concentration (*Ci*) of transgenic *PlPM19L* gene tobacco were higher than those of wild-type tobacco (Figure 7), and there was significant difference in *Gs*, *Tr,* and *Ci* between the transgenic *PlPM19L* and wild-type tobacco. In terms of values, the mean values of *Pn*, *Gs*, *Tr,* and *Ci* in transgenic *PlPM19L* tobacco were 1.06, 1.18, 1.17 and 1.18 times those in wild-type tobacco, respectively. These results indicated that transgenic *PlPM19L* tobacco plants were less vulnerable to drought stress.

### 2.9. Effects of Drought Stress on the Chlorophyll Fluorescence Parameters of Transgenic PlPM19L in N. tabacum

**Figure 8:**
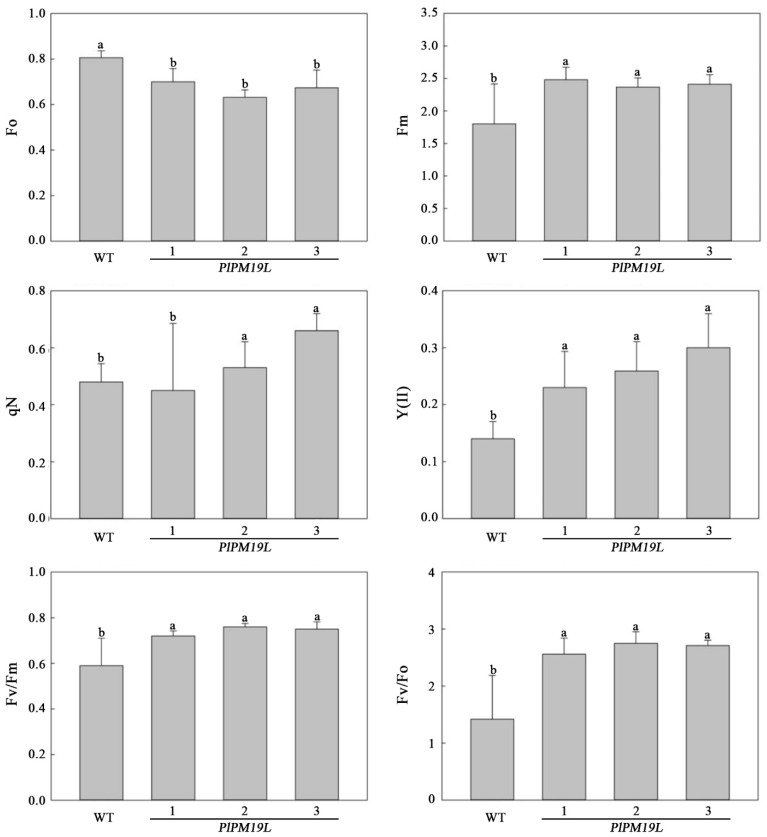
Chlorophyll fluorescence parameters of *PlPM19L* transgenic tobacco under drought stress. WT: wild-type tobacco; 1, 2 and 3: transgenic tobacco. Values are the means ± SE of three replicates. Different letters indicate significant differences (*p* < 0.05), and the same letters indicate no significant differences.

Chlorophyll fluorescence parameters can be used to determine the degree of damage to a plant photosystem under drought stress. A chlorophyll fluorescence spectrometer (Heinz Walz GmbH Effeltrich, Nebraska, Germany) was used to measure the chlorophyll fluorescence parameters of the wild-type and transgenic *PlPM19L* gene tobacco after they remained in the dark for 2 h. Compared with the wild-type tobacco, the maximum fluorescence (Fm), the maximum PS II photochemical efficiency (Fv/Fm), the PS II potential photochemical efficiency (Fv/Fo), and the actual photosynthetic efficiency of photosystem II (Y(II)) of each *PlPM19L* transgenic tobacco were significantly higher (Figure 8). However, the average value of non-photochemical quenching (qN) for the *PlPM19L* transgenic tobacco was higher than that of wild-type tobacco, and the minimal fluorescence (Fo) value of the *PlPM19L* transgenic gene tobacco was lower than that of wild-type tobacco.

## 3. Discussion

### 3.1. Structural Characteristics of PlPM19L and Its Expression Level in P. lactiflora

*PM19L*, a gene which participates in ABA synthesis and the signal transduction pathway, is regulated by ABA in plants, and it can also induce the ABA signaling pathway to participate in response to drought and high-salt, and low-temperature stress [20]. *PM19L* was first identified in wheat [22], and its orthologs have been identified from many plants such as *Oryza sativa* [20], *Arabidopsis thaliana* [21], and *Hordeum vulgare* [23]. The full-length cDNA sequence of *PlPM19L* in *P. lactiflora* was also identified and cloned in this study. We found that the full-length cDNA of *PlPM19L* was 910 bp encoding 178 amino acids, which was different from that of rice and wheat; their full lengths and number of encoded amino acids were 522 bp encoding 173 amino acids [20] and 985 bp encoding 181 amino acids [22], respectively.

Additionally, the relative expression levels of *PlPM19L* in the leaves of the control and treatment *P. lactiflora* ‘Dafugui’ were detected, and it was found that the expression level of *PlPM19L* was significantly up-regulated under drought stress. Additionally, many studies have shown that the *PM19L* gene can respond to drought, high salt levels, and low temperature by inducing the ABA signaling pathway [20,26]. In rice, the expression of *PM19L* was induced by heat shock, drought, cold, and salt stresses [20]. Therefore, it was speculated that drought stress may induce the gene expression of *PlPM19L* to promote the increase in ABA content in plant cells, and then activate some genes related to drought-stress response, inducing the stomatal closure of herbaceous peony, reducing transpiration, and effectively maintaining the water balance in the plant [27], and eventually defusing the damage caused to herbaceous peony under drought stress. Furthermore, the subcellular localization of the PlPM19L protein was observed in tobacco, and it was found that the PlPM19L protein was located on the cell membrane, which was consistent with the results observed in rice [20] and barley [23]. These results suggested that PlPM19L protein may be a membrane localization protein in the ABA signaling pathway, which may be related to the membrane and participate in ion transport, and may have a role in changing the membrane properties, thus regulating the response to drought stress [21].

### 3.2. Phenotype and ABA Content of PlPM19L Transgenic N. tabacum

In order to further study the mechanism of *PlPM19L* in response to drought stress, *PlPM19L* was transferred into model plant tobacco, and wild-type tobacco and transgenic tobacco were treated with natural drought stress at the same time. After 10 days of drought stress, wild-type tobacco leaves showed obvious wilting and drooping, while the *PlPM19L* transgenic tobacco leaves continued to maintain a normal growth state (Figure 5A). Many studies have shown that drought stress can cause changes in plant morphology and other physiological activities, such as the decrease in relative water content in leaves, the increase in protective enzyme activity, and the weakening of the photosynthetic apparatus and photosynthetic efficiency. Drought stress can affect and damage plants in varying degrees [28]. In this study, compared with wild-type tobacco, the relative water content of leaves from *PlPM19L* transgenic tobacco was significantly higher, indicating that the overexpression of *PlPM19L* in tobacco could effectively reduce the rate of water loss, reduce the intensity of cell membrane damage, and then improve the drought resistance of tobacco. Similarly, the ABA content of *PlPM19L* transgenic tobacco was higher than that of wild-type tobacco under drought stress (Figure 5C). Therefore, it was speculated that the *PlPM19L* gene in tobacco induced the ABA signaling pathway, promoted an increase in ABA content, and then enhanced the drought tolerance of plants, which was consistent with the results of a study regarding *OsPM19L* in rice [20,29]. As an important hormone, ABA plays an important role in plant growth, stomatal movement, seed dormancy, and resistance and adaptation to drought stress [30]. The content of ABA in herbaceous peony leaves increased with more acute drought stress, and the content of ABA in the stronger drought-resistance variety was higher than that in the weaker drought-resistance variety [31]. Studies indicated that an increase in ABA content was due to osmotic stress in plants subjected to abiotic stresses, such as drought stress, salinity, and cold [32]. It is well known that an appropriate level of ABA is required for successful plant growth under adverse conditions [33]. Under drought stress, ABA is important in signaling by enabling stomatal closure, reducing transpiration, and maintaining growth [34]. Therefore, compared with the wild-type tobacco, the *PlPM19L* transgenic tobacco plants had higher ABA content, which made the plants more drought resistant.

### 3.3. Photosynthesis of PlPM19L Transgenic N. tabacum

Drought stress will reduce the activity of protective enzymes in plants, lead to the accumulation of ROS in plants, resulting in the destruction of chlorophyll [35], and then reduce the photosynthetic characteristics and chlorophyll fluorescence parameters [36]. Antioxidant enzymes such as CAT, SOD, POD and APX either scavenge the toxic ROS or defend plants by activating a non-enzymatic antioxidant system [37]. In our previous research, the antioxidant enzyme activity in *P. lactiflora* leaves changed significantly under drought stress [8]. In this study, the enzyme activity levels of SOD, POD, CAT, and APX in *PlPM19L* transgenic tobacco were higher than those in wild-type tobacco, indicating that the overexpression of the *PlPM19L* gene could more effectively increase the activity of protective enzymes to remove ROS. Drought affects photosynthesis, and photosynthetic rates decline under drought stress [38]. Normally, chlorophyll fluorescence is used to study the overall photosynthetic process in plants [39]. The net *Pn*, *Gs*, *Ci*, and *Tr* in plants decreased under drought stress, with the extent of the decrease varying among plants [40]. Similarly, our previous research has shown that the *Pn*, *Gs*, and *Tr* in herbaceous peony leaves under drought stress decreased significantly [8]. Among the related indicators of plant photosynthetic characteristics, *Pn* can directly reflect the strength of leaf photosynthetic capacity, *Gs* can reflect the ability of plants to regulate water consumption, *Ci* directly affects the net photosynthetic rate of plants, and *Tr* can be used to reflect the ability of water metabolism in plants [41]. In this study, the *Pn*, *Gs*, *Ci*, and *Tr* levels in the *PlPM19L* transgenic tobacco plants were higher than those in the wild-type tobacco, which showed that the transgenic plants had stronger photosynthetic capacity and higher water use efficiency compared with wild tobacco. This indicated that *PlPM19L* transgenic tobacco plants had strong resistance to drought stress. In addition, chlorophyll fluorescence parameters can reflect the damage to the photosynthetic reaction and photosynthetic apparatus and can be used as internal probes to investigate the response of plant photosynthesis to various abiotic stress [42]. Previous studies have shown that chlorophyll fluorescence parameters, including Fo, Fv, Fm, Fv/Fm, Fv/Fo, Y(II), qN, etc. were related to drought stress in plants [38,42,43]. Likewise, compared with the control, the Fv/Fm, Fv/Fo and Y(II) in herbaceous peony under drought stress were significantly decreased [8,44]. In this study, the Fm, qN, Y(II), Fv/Fm, and Fv/Fo values in the *PlPM19L* transgenic tobacco plants were higher than those in the wild-type tobacco, while the Fo value in the *PlPM19L* transgenic tobacco plants was lower than that in the wild-type tobacco. Among them, Fv/Fm is regarded as an important indicator of the PSII photoinhibition of plants with various abiotic stress stresses, and it indicates the maximal photochemical efficiency of PSII and also indicates functional loss of the PSII action center [38]. When plants are under non-adversity conditions, the Fv/Fm value is generally between 0.75 and 0.85, and it is related to the quantum yield of photochemistry [38]. However, in this study, although the *PlPM19L* transgenic tobacco was treated by drought stress, the Fv/Fm value was still around 0.75, indicating that drought stress cause little damage to the PSII action center of the *PlPM19L* transgenic tobacco, which could be used to capture light energy well for photosynthesis and greatly improve the photosynthetic efficiency.

## 4. Material and Method

### 4.1. Plant Materials and Treatment

Five-year-old *Paeonia lactiflora* ‘Da Fugui’, which were cultivated in potting soil (loam: peat: coarse sand, 1:1:1) with similar initial growth statuses, were divided into two groups. One group acted as a control group and was watered everyday at 17:00, and another group was not watered and was subjected to natural drought stress, which was the treatment group. The leaves of the plants in the treatment group and the control group were collected on 0, 7, 14, and 21 days after treatment and stored at −80 °C for RNA extraction.

The transformed plant materials were the leaves of *N. tabacum.* Wild-type tobacco and transgenic tobacco were cultivated for two months and fully watered before undergoing drought stress. The phenotypes of tobacco were significantly different after drought stress treatment for about 10 days, and the physiological indexes, photosynthetic characteristics, and chlorophyll fluorescence of the leaves were measured. Additionally, the remaining leaves were frozen with liquid nitrogen and stored at −80 °C for future use.

### 4.2. Gene Cloning and Sequence Analysis

After the leaves were ground into powders, RNA was separately extracted using the Mini BEST Plant RNA Extraction Kit (TaKaRa, Tokyo, Japan) accoridng to the manufacturer’s instructions, genomic DNA (gDNA) was removed by Recombinant DNase I, and all RNA samples were examined with Nanodrop 2000C (Thermo Scientific, Waltham, MA, USA). The full-length cDNA of *PlPM19L* was isolated via RACE technology using a SMART RACE cDNA Amplification Kit (TaKaRa, Tokyo, Japan) according to the manufacturer’s instructions. The specific primers used for 5’/3’ RACE PCR amplification were designed by Primer 5.0 software (Table S1). After being tested via 1% (*w*/*v*) agarose gel electrophoresis, the *PlPM19L* PCR products were cloned into the pClone007 Vector and sequenced.

The amino acid composition, relative molecular weight, isoelectric point, and other physical and chemical properties of *PlPM19L* were analyzed via Protparam (http://web.expasy.org/protparam) (accessed on 17 September 2022). The protein sequences of the other plants were obtained from the NCBI, and the sequence alignment was analyzed via DNAMAN. A neighbor-joining phylogenetic tree was generated with MEGA 7.0, and bootstrap values were set as 1000 bootstrap replicates [45].

### 4.3. Gene Expression Analysis Using Quantitative Real-Time PCR (qRT-PCR)

qRT-PCR was introduced to analyze the gene transcript levels with BIO-RAD CFX Connect^TM^ Optics Module (Bio-Rad, Hercules, CA, USA). Leaves’ extracted RNA (1 μg) was reverse-transcribed into cDNA in a 10 μL reaction based on the superscript first-strand synthesis system (PrimeScript^®^ RT Reagent Kit With gDNA Eraser, TaKaRa, Tokyo, Japan). *PlActin* (JN105299) and *NtActin*(AB158612) were used as internal reference genes. All of the specific primers for qRT-PCR were synthesized by Shanghai Sangon Biological Engineering Technology and Services Co., Ltd., (Shanghai, China) and the sequences are shown in Table S2. SYBR^®^ Premix Ex Taq^TM^ (Perfect Real Time) was used for qRT-PCR (TaKaRa, Tokyo, Japan). The PCR cycles were as follows: 55 °C for 2 min, followed by an initial denaturation step at 95 °C for 30 s, 40 cycles at 95 °C for 5 s, 55 °C for 15 s, and 72 °C for 30 s. The 2^−ΔΔCt^ comparative threshold cycle (Ct) method was used for the calculation of relative expression levels [46].

### 4.4. Construction of Expression Vector

After the PCR amplification of the PlPM19L-Forward Primer and PlPM19L-Reverse Primer (synthesized by Nanjing Qingke Biotechnology Co., Ltd., Nanjing, China), the electrophoretic bands were cut off, and then we used the TSINGKE Gel DNA GEL Extraction Kit to recover the PCR products and link product and the carrier. The vector fragment, *pBWA(V)HS-GLosgfp*, and the amplified fragment, PlPM19L, were subjected to enzyme digestion reactions. After 1 h of reaction at 37 °C, the digested products were ligated at 16 °C for 2 h with T4 DNA ligase. The product was transformed into Escherichia coli, coated on the medium containing Kana resistance, and incubated at 37 °C for 8 h; then, the plaque was picked for PCR reaction. The bacterial liquid corresponding to the electrophoresis band in the correct position was selected for sequencing, and the plasmid was extracted from the bacterial liquid with the correct sequence and verified via enzyme digestion.

### 4.5. Subcellular Localization

The coding sequence of *PlPM19L* without a stop codon was amplified via gene-specific primers (forward 5′-CAGTGGTCTCACAACATGGCTGCAGTTGGGAAGAC-3′; reverse 5′-CAGTGGTCTCATACAAATCCTAGTGCCAGTGTCAC-3′) and was fused into the green fluorescent protein (GFP) region of the *pBWA(V)HS-GLosgfp* vector. Then, the fusion constructs of *pBWA(V)HS-PlPM19L-GLosgfp* and empty *pBWA(V)HS-GLosgfp* were transformed into *Agrobacterium tumefaciens* strain *GV3101* via the freeze–thaw method. Overnight *Agrobacterium* cultures containing *pBWA(V)HS-PlPM19L-GLosgfp* and empty *pBWA(V)HS-GLosgfp* vectors were resuspended in resurrection buffer (10 mM MES, 10 mM MgCl_2_, and 0.2 mM acetosyringone) and then were injected into 4~5-week-old *Nicotiana benthamiana* leaves. After 2 days of weak light cultivation, the GFP signals of *pBWA(V)HS-PlPM19L-GLosgfp* and empty *pBWA(V)HS-GLosgfp* were observed via confocal laser microscopy (Nikon C2-ER, Tokyo, Japan) to determine the subcellular localization of *PlPM19L*.

### 4.6. Overexpression of PlPM19L in the N. tabacum

The overexpression vectors were transformed into *Agrobacterium tumefaciens* strain GV3101 as above. *A. tumefaciens* cultures containing *PlPM19L* were transformed into tobacco ‘K326’ using the leaf disc method [47].

### 4.7. Measurement of Relative Water Content

An electronic balance was used to weigh an appropriate number of fresh leaves, and the weight was recorded as fresh weight (FW). The leaves were treated in an oven at 105 °C for 5 min, and then the temperature was adjusted to 65 °C for 2 h. The sample with constant weight was weighed and recorded as dry weight (DW). The relative water content (%) was calculated as (FW-DW)/FW × 100%.

### 4.8. Measurement of ABA Content

ABA content measurement was performed using the enzyme linked immunosorbent assay (ELISA) method. The specific method was according to the Reagent Kit of ABA (ELISA) (Shanghai Sinobest Biological Science and Technology Ltd., Shanghai, China), as described below. Briefly, 0.1 g fresh leaf samples were homogenized with 1 mL of phosphate-buffered saline, and then, the samples were centrifuged at 10,000 rpm for 20 min to obtain the supernatant. For ELISA, the supernatant and ABA antibody were kept in the microplate and incubated at 37 °C for 1 h, After washing the microplate 5 times, the substrate was added and incubated at 37 °C for 15 min. The ABA content was determined by the SpectraMax M5 plate reader (Molecular Devices Corporation, Sunnyvale, CA, USA) by measuring absorbance at 450 nm, and the spectrophotometric values were substituted into the standard curve to calculate the ABA contents. Three biological repeats were performed.

### 4.9. Accumulation Level of H_2_O_2_

The accumulation level of H_2_O_2_ was measured via diaminobenzidine (DAB) staining [44]. A 0.1 mg/mL diaminobenzidine (DAB) staining solution with pH 5.0 was prepared with 50 mM Tris-acetate buffer. The fresh leaves were fully immersed in the DAB staining solution for 24 h in the dark. Then, the soaked leaves were placed into 95% alcohol to be boiled. After 15 min, they were photographed, and the accumulation of H_2_O_2_ was observed.

### 4.10. Measurement of Antioxidant Enzyme Activities

Superoxide dismutase (SOD), peroxidase (POD), catalase (CAT), and ascorbate peroxidase (APX) activity measurements were all performed using the UV spectrophotometer method. The specific methods were according to the Reagent Kit of SOD, POD, CAT, and APX (Suzhou Keming Biotechnology Co., Ltd., Suzhou, China), respectively.

### 4.11. Measurement of Photosynthetic Characteristics

The photosynthetic characteristics were measured with a portable photosynthesis system (LI-6400, Li-Cor, Lincoln, NE, USA) at 8:00 to 9:00 a.m. local time. The standard leaf chamber with a 2 cm × 3 cm photosynthetic photon quanta flux density (PPFD) was set at 1000 μmol·m^−2^·s^−1^ using a self-taking red and blue light-emitting diode (LED) source. The net photosynthesis rate (Pn), transpiration rate (Tr), stomatal conduction (Gs), and intercellular CO_2_ concentration (Ci) were also recorded in the system.

### 4.12. Measurement of Chlorophyll Fluorescence Parameters

The chlorophyll fluorescence parameters were measured using the chlorophyll fluorescence spectrometer (Heinz Walz GmbH, Effeltrich, Nebraska, Germany) at 21:00 to 22:00 PM local time after the tobaccos had remained in the dark for 2 h. This system recorded the minimal fluorescence (Fo), the maximum fluorescence (Fm) and the variable fluorescence (Fv), while the maximum PS ‖ photochemical efficiency (Fv/Fm), the non-photochemical quenching (qN), the actual photosynthetic efficiency of photosystem II (Y(II)), the PS II potential photochemical efficiency (Fv/Fo) were calculated via PAM Win, the data processing software of this instrument. 

### 4.13. Statistical Analysis

All the data were recorded by means of three replicates with standard deviation. The results were analyzed for variance using the SAS/STAT statistical analysis package (Version 6.12, SAS Institute, Cary, NC, USA) and drawn with SigmaPlot 12.5.

## 5. Conclusions

In the present study, we demonstrated that *PlPM19L* is a key factor in increasing the content of ABA to confer drought stress tolerance. The overexpression of *PlPM19L* ameliorates H_2_O_2_ accumulation, increases antioxidant enzyme activity levels, and increases the potential photosynthetic capacity under drought stress, all of which could provide a valuable foundation for breeding drought-resistant *P. lactiflora* varieties and allowing economically important plants to be grown in areas where they are subjected to multiple kinds of abiotic stress.

## Data Availability

Data are contained within the article or Supplementary Files.

## Supplementary Materials

The supporting information can be downloaded at: https://www.mdpi.com/article/10.3390/ijms232415695/s1.

## References

[1] Qing K.J. (2004). Illustration of One Hundred Ornamental Flowers Bonsai—The Herbaceous Peony.

[2] Zhao D.Q., Luan Y.T., Xia X., Shi W.B., Tang Y.H., Tao J. (2020). Lignin provides mechanical support to herbaceous peony (*Paeonia lactiflora Pall*.) stems. Hortic. Res..

[3] Meng J.S., Tang Y.H., Sun J., Zhao D.Q., Zhang K.L., Tao J. (2021). Identification of genes asscociated with the biosynthesis of unsaurated fatty acid and oil accumulation in herbaceous peony ‘Hangshao’ (Paeonia lactiflora ‘Hangshao’) seeds based on transcriptome analysis. BMC Genom..

[4] Sun J., Guo H.X., Tao J. (2022). Effects of harvest stage, storage, and preservation technology on postharvest ornamtal value of cut peony (Paeonia lactilfora) flowers. Agronomy.

[5] Kamenetsky R., Dole J. (2012). Herbaceous peony (*Paeonia*): Genetics, physiology and cut flower production. Floric. Ornam. Biotechnol..

[6] Zhang W.T., Yang X.J., Tang D.Q. (2011). Study on plant hardiness and heat zones in China. Chin. Landsc. Archit..

[7] Zhang J.P., Zhang D., Wei J.F., Shi X.H., Ding H.Q., Qiu S., Guo J., Li D.Q., Zhu K.Y., Horvath D.P. (2019). Annual growth cycle observation, hybridization and forcing culture for improving the ornamental application of *Paeonia lactiflora* Pall. in the low-latitude regions. PLoS ONE.

[8] Li T.T., Wang R., Zhao D.Q., Tao J. (2020). Effect of drought stress on physiological responses and gene expression changes in herbaceous peony (*Paeonia lactiflora* Pall.). Plant Signal. Behav..

[9] Huang J.P., Ma J.R., Guan X.D., Li Y., He Y.L. (2019). Progress in semi-arid climate change studies in China. Adv. Atmos. Sci..

[10] Qin Y.W., Liu J.Y., Shi W.J., Tao F.L., Yan H.M. (2013). Saptial-temporal changes of cropland and climate potential productivity in northern China during 1990–2010. Food Secur..

[11] Soma F., Takahshi F., Yamaguchi-Shinozaki K., Shinozaki K. (2021). Celluar phophorylation signaling and gene expression in drought stress responses: ABA-dependent and ABA-independent regulatory system. Plants.

[12] Zhao D.Q., Luan Y.T., Shi W.B., Zhang X.Y., Meng J.S., Tao J. (2021). A *Paeonia ostia* caffeoyl-CoA *O*-methyltransferase confers drought stress tolerance by promoting lignin synthesis and ROS scavenging. Plant Sci..

[13] Yoshida T., Fujita Y., Maruyama K., Mogami J., Todaka D., Shinozaki K., Yamaguchi-Shinozaki K. (2015). Four Arabidopsis AREB/ABF transciption factors function predominantly in gene expressioin downstrean of SnRK2 kinases in abscisic signalling in response to osmotic stress. Plant Cell Environ..

[14] Kidokoro S., Watanabe K., Ohori T., Moriwaki T., Maruyama K., Mizoi J., Myint N., Htwe P.S., Fujita Y., Sekita S. (2015). Soybean DREB1/CBF-type transcription factors function in heat and drought as well as cold stress-responsive gene expression. Plant J..

[15] Chen S.J., Xu K., Kong D.Y., Wu L.Y., Chen Q., Ma X.S., Li T.F., Xie Q., Liu H.Y., Luo L.J. (2022). Ubiquitin ligase OsRINGzf1 regulates drought resistance by controlling the turnover of OsPIP2;1. Plant Biotechnol. J..

[16] Sallam A., Alqudah A.M., Dawood M.F.A., Baenziger P.S., Börner A. (2019). Drought stress tolerance in wheat and barley: Advances in physiology, breeding and genetic research. Int. J. Mol. Sci..

[17] Takahashi F., Kuromori T., Sato H., Shinozaki K. (2018). Regulatory gene networks in drought stress responses and resistance in Plants. Adv. Exp. Med. Biol..

[18] Kar R.K. (2011). Plant responses to water stress: Role of reactive oxygen species. Plant Signal. Behav..

[19] Postinglion A.E., Muday G.K. (2020). The role of ROS homeostasis in ABA-induced guard cell signaling. Front. Plant Sci..

[20] Chen H., Lan H., Huang P., Zhang Y., Yuan X., Huang X., Huang J., Zhang H. (2015). Characterization of *OsPM19L1* encoding an AWPM-19-like family protein that is dramatically induced by osmotic stress in rice. Genet. Mol. Res..

[21] Barrero J.M., Dorr M.M., Talbot M.J., Ishikawa S., Umezawa T., White R.G., Gubler F. (2019). A role for *PM19-Like 1* in seed dormancy in *Arabidopsis*. Seed Sci. Res..

[22] Koike M., Takezawa D., Arakawa K., Yoshida S. (1997). Accumulation of 19-kDa plasma membrane polypeptide during induction of freezing tolerance in wheat suspension-cultured cells by abscisic acid. Plant Cell Physiol..

[23] Ranford J.C., Bryce J.H., Morris P.C. (2002). PM19, a barley (*Hordeum vulgare* L.) gene encoding a putative plasma membrane protein, is expressed during embryo development and dormancy. J. Exp. Bot..

[24] Zhao D.Q., Tao J., Han C.X., Ge J.T. (2012). Actin as an alternative internal control gene for gene expression analysis in herbaceous peony (*Paeonia lactiflora* Pall.). Afr. J. Agr. Res..

[25] Weaver J., Goklany S., Rizvi N., Cram E.J., Lee-Parsons C.W. (2014). Optimizing the transient Fast Agro-mediated Seedling Transformation (FAST) method in *Catharanthus roseus* seedling. Plant Cell Rep..

[26] Rerksiri W., Zhang X., Xiong H., Chen X. (2013). Expression and promoter analysis of six heat stress-inducible genes in rice. Sci. World J..

[27] Shinozaki K., Yamaguchi-Shinozaki K. (2006). Gene networks involved in drought stress response and tolerance. J. Exp. Bot..

[28] Hura T., Hura K., Ostrowska A. (2022). Drought-stress induced physiological and molecular changes in plants. Int. J. Mol. Sci..

[29] Yao L.Y., Cheng X., Gu Z.Y., Huang W., Li S., Wang L.B., Wang Y.F., Xu P., Ma H., Ge X.C. (2018). The AWPM-19 family protein OsPM1 mediates abscisic acid influx and drought response in rice. Plant Cell.

[30] Seo M., Marion-Poll A. (2019). Abscisic acid metabolism and transport. Adv. Bot. Res..

[31] Wang Q., Zhao R., Chen Q.H., da Silva J.A.T., Chen L.Q., Yu X.N. (2018). Physiological and biochemical responses of two herbaceous peony cultivars to drought stress. HortScience.

[32] Nguyen T.T.Q., Trinh L.T.H., Pham H.B.V., Le T.V., Phung T.K.H., Lee S.K., Cheong J.J. (2020). Evaluation of proline, soluble sugar and ABA content in soybean *Glycine max* (L.) under drought stress memory. AIMS Bioeng..

[33] Spollen W., LeNoble M., Samuels T., Bernstein N., Sharp R. (2000). Abscisic acid accumulation maintains maize primary root at low water potentials by restricting ethylene production. Plant Physiol..

[34] Cenzano A.M., Reginato M., Varela M.C., Luna M.V. (2018). Abscisic acid and its metabolites are involved in drought tolerance in four native species of Patagonian semiarid shrublands (Argentina). Aust. J. Bot..

[35] Uenyayar S., Keles Y., Cekic F.O. (2005). The antioxidative response of two tomato species with different drought tolerances as a results of drought and cadmium stress combinations. Plant Soil Environ..

[36] Zhao J., Lang Y., Zhang S.Y., Zhao Q.K., Zhang C.Y., Xia J.B. (2019). Photosynthetic characteristics and chlorophyll a fluorescence transient in *Lonicera japonica* under drought stress. Acta Physilogogiae Plant..

[37] Meng J.S., Li M., Hao Z.J., Zhao D.Q., Tao J. (2022). Paclobutrazol can enhance the themar-taolerant on herbaceous peony (*Paeonia lactiflora*). Russ. J. Plant Physiol..

[38] Simeneh A.T. (2020). Photosynthesis limiting stresses under climate change scenarios and role of chlorophyll fluorescence: A review article. Cogent Food Agric..

[39] Murchie E.H., Lawson T. (2013). Chlorophyll fluorescence analysis: A guide to good practice and understandings new applications. J. Exp. Bot..

[40] Ma L., Zhang Z.H., Yao B.Q., Ma Z., Huang X.T., Zhou B.R., Xu M.H., Guo J., Zhou H.K. (2021). Effects of drought and heat on the productivity and photosynthetic characteristics of alpine meadow plants on the Qinghai-Tibetan Plateau. J. Mt. Sci..

[41] Zhang Q., Li P., Tian J., Li J. (2019). Study on Photosyntheic and chlorophyll fluorescence characteristics of transgenic tobacco with *AcCBF1* gene. Xinjiang Agric. Sci..

[42] Çiçek N., Pekcan V., Arslan Ö., Erdal Ş.Ç., Nalçaiyi A.S.B., Çil A.N., Şahin V., Kaya Y., Ekmekçi Y. (2019). Assessing drought tolerance in field-grown sunflower hybrids by chlorophyll fluorescence kinetics. Braz. J. Bot..

[43] Killi D., Raschi A., Bussotti F. (2020). Lipid peroxidation and chlorophyll fluorescence of photosystem II performance during drought and heat stress in associated with the antioxidant capacities of C3 sunflower and C4 maize varieties. Int. J. Mol. Sci..

[44] Zhao D.Q., Zhang X.Y., Wang R., Liu D., Sun J., Tao J. (2019). Herbaceous peony tryptophan decarboxylase confers drought and salt stresses tolerance. Environ. Exp. Bot..

[45] Saitou N., Nei M. (1987). The neighbor-joining method: A new method for reconstructing phylogenetic tress. Mol. Biol. Evol..

[46] Schmittgen T.D., Livak K.J. (2008). Analyzing real-time PCR data by the comparative CT method. Nat Protoc..

[47] Sunilkumar G., Vijayachandra K., Veluthambi K. (1999). Preincubation of cut tobacco leaf explants promotes *Agrobacterium*-mediated transformation by increasing vir gene induction. Plant Sci..

