# Supplementary material for: Analysis and Functional Verification of PlPM19L Gene Associated with Drought-Resistance in Paeonia lactiflora Pall
Source: Int J Mol Sci. 2022 Dec 10;23(24):15695. doi: 10.3390/ijms232415695 (PMC9779317; doi:10.3390/ijms232415695)
Supplement: Supplementary file 1 [file ijms-23-15695-s001.zip › Table S1.pdf]

Table S1. Gene-specific primers sequence for RACE amplification of *PM19L* gene

Primers	Sequence(5'-3')	Amplification
3'-*PM19L* Outer	CTCTTGTATGTCTTGTTGCTTC	1st of 3'-RACE
3'-*PM19L* Inner	GTCTGTTCAGCAGCAAGT	2nd of 3'-RACE
5'-*PM19L*	GCAGTGATTGCCCATGCTATGAGTGAG	5'-RACE
