# Supplementary material for: Analysis and Functional Verification of PlPM19L Gene Associated with Drought-Resistance in Paeonia lactiflora Pall
Source: Int J Mol Sci. 2022 Dec 10;23(24):15695. doi: 10.3390/ijms232415695 (PMC9779317; doi:10.3390/ijms232415695)
Supplement: Supplementary file 1 [file ijms-23-15695-s001.zip › Table S2.pdf]

Table S2. Gene-specific primers sequence for qRT-PCR

Gene	Forward primer（5' - 3'）	Reverse primer（5' - 3'）
PlActin	GTTGCCCTTGATTACGAG	GCTTCCATTCCGATTAGTG
PlPM19L	GGAAAAGATTTGAAGATGGC	TTGCTGCTGAACAGACCC
NtActin	TCCTCATGCAATTCTTCG	ACCTGCCCATCTGGTAAC
